# University students' perceptions of virtuous persons in Türkiye: associations with psychological wellbeing, resilience, intolerance of uncertainty, and hopelessness

**DOI:** 10.3389/fpsyg.2026.1961607

**Published:** 2026-10-16

**Authors:** Şener Şentürk, Duygu Gür Erdoğan, Mustafa Selim Altınışık, Fadıl Şiraz, Kevser Kara

**Affiliations:** 1 Department of Educational Sciences, Faculty of Education, Ondokuz Mayis University, Samsun, Türkiye; 2 Department of Educational Sciences, Faculty of Education, Sakarya University, Sakarya, Türkiye; 3 Gaziantep Provincial Directorate of National Education, Ministry of National Education, Gaziantep, Türkiye

**Keywords:** hopelessness, intolerance of uncertainty, moral evaluation, perceptions of virtuous persons, psychological resilience, psychological wellbeing, university students

## Abstract

**Introduction:**

Perceptions of virtuous persons represent an important yet understudied dimension of moral cognition that may be associated with individuals' psychological functioning as well as their developmental and contextual experiences. This study examined university students' perceptions of virtuous persons in relation to demographic characteristics, psychological wellbeing, psychological resilience, intolerance of uncertainty, and hopelessness.

**Methods:**

This cross-sectional correlational study included 1,322 undergraduate students aged 18–32 years enrolled at five universities in Türkiye. Exploratory demographic differences were examined using an independent-samples *t*-test and one-way analyses of variance, while the unique contributions of the psychological variables were assessed using multiple linear regression.

**Results:**

The regression model was statistically significant and explained 35.9% of the variance in perceptions of virtuous persons. Psychological wellbeing was the strongest positive predictor (β = 0.580, *p* < 0.001). Intolerance of uncertainty also showed a smaller positive association (β = 0.138, *p* < 0.001), contrary to the hypothesized negative direction. Psychological resilience and hopelessness did not make significant unique contributions. Exploratory analyses also identified differences according to gender, income level, year of study, and academic achievement, although the effects were modest.

**Discussion:**

The findings indicate that psychological characteristics are not uniformly associated with perceptions of virtuous persons and that psychological wellbeing accounts for most of the model's explanatory contribution. The unexpected positive association between intolerance of uncertainty and perceptions of virtuous persons warrants cautious interpretation. Longitudinal and cross-cultural research is needed to clarify the direction and underlying mechanisms of these associations.

## 1 Introduction

Understanding human behavior solely through biological mechanisms or environmental determinants may yield a reductionist interpretation of complex psychological and moral processes (Kunkel, 2021; Holahan, 2012). Individuals' evaluative judgments regarding what is good, right, or desirable constitute a central explanatory dimension of behavior (Kristiansen and Hotte, 2013; Sparks and Shepherd, 2002). Within this broader moral-psychological framework, the concept of virtue emerges as a foundational construct for understanding how individuals orient themselves toward value-based action. In recent decades, advances in positive psychology have increasingly conceptualized virtue as a psychological resource that contributes to human flourishing rather than merely as a philosophical or ethical ideal (Peterson and Seligman, 2004; Kesebir and Diener, 2014). Within this perspective, virtues are regarded as character strengths that facilitate adaptive psychological functioning by promoting wellbeing, prosocial behavior, and healthy interpersonal relationships (Peterson and Seligman, 2004; Niemiec, 2018). This theoretical development has also shifted scholarly attention from defining virtue itself toward understanding how individuals perceive and mentally represent virtuous persons. From a theoretical perspective, conceptions of virtue may provide evaluative frameworks through which individuals interpret moral character and morally desirable conduct (Kristjánsson, 2015; Moberg, 2000).

As a multidimensional psychological construct, virtue encompasses a constellation of character strengths that guide individuals toward morally and socially desirable patterns of thought, emotion, and behavior (Peterson and Seligman, 2004; Sentürk et al., 2024; Yigitce and Sentürk, 2024).

In general terms, virtue can be understood as an individual's inclination toward what is morally good and the capacity to consistently enact behaviors aligned with this inclination (Turan, 2015). This perspective positions virtue not merely as a moral abstraction but as a dynamic structure that integrates cognition, affect, and behavioral consistency.

Values such as justice, honesty, responsibility, compassion, generosity, patience, and self-control function as an internal regulatory compass guiding individuals' actions. This compass does not merely indicate what is normatively desirable; it also supports the persistence and coherence of morally congruent behavior over time. Accordingly, virtuous conduct is often characterized by consideration of others' interests, adherence to ethical principles, and a sense of social responsibility (Jacobson, 2005; Fortenbaugh, 1964). These interrelated qualities suggest that virtue represents a multidimensional constellation of compatible value orientations rather than a single dispositional trait (Williams, 2018; Mitchell, 2015). From the perspective of positive psychology, virtues are not merely desirable moral attributes but also enduring psychological capacities that enable individuals to regulate emotions, establish meaningful interpersonal relationships, and pursue valued life goals. By fostering adaptive coping, empathy, and prosocial engagement, virtues contribute to psychological resilience and overall wellbeing, highlighting their role as important psychological resources rather than solely ethical ideals (Peterson and Seligman, 2004; Niemiec, 2018; Kesebir and Diener, 2014). Taken together, the theoretical perspectives reviewed above suggest that virtue represents a psychologically organized system of values through which individuals interpret themselves, evaluate others, and regulate their social interactions. Consequently, examining individuals' perceptions of virtuous persons may provide valuable insights into the cognitive and affective mechanisms underlying moral judgments and value-based social evaluations.

Importantly, examining virtue solely as an individual characteristic may overlook a critical dimension: how individuals cognitively and affectively construct the image of a virtuous person. Rather than functioning merely as descriptions of desirable characteristics, perceptions of virtuous persons may influence how individuals interpret social information, evaluate others, and organize their moral understanding (Walker and Frimer, 2007; Kristjánsson, 2015). These perceptions are shaped by personal values, social learning experiences, and broader psychological functioning, making them psychologically meaningful rather than merely descriptive judgments. As such, they serve as normative reference points that influence role-model selection, social affiliation patterns, and behavioral regulation (Moberg, 2000). Consequently, investigating individuals' perceptions of virtuous persons provides valuable insights into the cognitive and affective mechanisms through which moral judgments and value-based social evaluations are formed.

University students constitute a theoretically relevant population for examining perceptions of virtuous persons because higher education represents not only an academic setting but also a formative social context in which students encounter diverse values, identities, relationships, and ethical perspectives. University students regularly make value-based judgments within academic collaborations, peer relationships, professional preparation, and decisions concerning their future social roles. These experiences may encourage students to reconsider the characteristics they associate with morally admirable individuals and to refine the standards they use when evaluating themselves and others. Moreover, for many students, the university years overlap with emerging adulthood, a period characterized by identity exploration, increasing autonomy, and the consolidation of personal values (Arnett, 2000, 2007; Torres et al., 2009). Nevertheless, the relevance of undergraduate students as the study population does not rest solely on their developmental stage. The higher education environment itself offers repeated opportunities for identity negotiation, ethical reflection, exposure to diverse perspectives, and observation of potential role models (Carr, 2017; Gyamera, 2018; Scanlon et al., 2007).

Within this educational and social context, students evaluate others and regulate their own conduct across friendships, romantic relationships, academic collaborations, and emerging professional roles. Recent research has similarly connected virtue-related characteristics with the quality of interpersonal relationships, moral identity, and prosocial behavior among university students and emerging adults (Daniilidou and Nerantzaki, 2025; Liu et al., 2025). Perceptions of virtuous persons may therefore operate as evaluative reference points that influence how students recognize desirable personal characteristics, select role models, and interpret interpersonal conduct. University students also encounter uncertainty associated with academic demands, career decisions, relationships, and future social roles (Arnett, 2007; Kong and Zeng, 2023). However, students exposed to similar educational and social circumstances may differ in how they recognize, prioritize, and evaluate virtuous characteristics, suggesting that individual psychological functioning may contribute to these perceptions.

The development of virtue-related perceptions is influenced by multiple contextual factors, including family socialization, role models, educational experiences, and broader cultural norms (Lickona, 2013; Moberg, 2000). During the university years, peer relationships, academic environments, and expanding social experiences may further contribute to the formation and refinement of value systems (Ossowska, 2016; Vallor, 2012). Nevertheless, contextual influences alone may not fully explain individual differences in perceptions of virtuous persons. Based on their established associations with psychological functioning, psychological wellbeing and resilience were expected to be positively associated with perceptions of virtuous persons, whereas intolerance of uncertainty and hopelessness were expected to show negative associations. Because previous research has not directly examined these variables in relation to perceptions of virtuous persons, the proposed relationships were treated as theoretical expectations rather than established empirical associations. Accordingly, the present study examined the unique associations of these four psychological characteristics with university students' perceptions of virtuous persons within a single predictive model.

As a core indicator of positive psychological functioning, psychological wellbeing reflects individuals' capacity to maintain a meaningful, purposeful, and psychologically integrated life while establishing positive relationships with others (Ryff and Singer, 1996). Such characteristics may facilitate more adaptive perceptions of virtuous persons by enhancing individuals' capacity for meaning-making, positive interpersonal engagement, and balanced moral evaluation. Individuals with higher levels of psychological wellbeing tend to experience greater psychological integration and more positive orientations toward interpersonal relationships, enabling them to evaluate others through more constructive and value-consistent cognitive frameworks (Ryff and Singer, 1996; Kesebir and Diener, 2014). Consequently, they may be more inclined to recognize virtuous characteristics as meaningful, attainable, and personally relevant, thereby developing more adaptive perceptions of virtuous persons.

Psychological resilience refers to the capacity to maintain or regain adaptive functioning in the face of adversity (Rutter, 1985; Sisto et al., 2019). Research on resilience has examined its links with mental health and adaptive responses to stressful experiences (Davydov et al., 2010). Tugade et al. (2004) further showed that positive emotions play a role in how resilient individuals cope with negative events. These findings provide a basis for considering whether resilience is associated with evaluations of other people; they do not, however, demonstrate an association with perceptions of virtuous persons. In the present study, we therefore examine the theoretically proposed association between psychological resilience and perceptions of virtuous persons, without presuming that coping or emotional processes explain it. Intolerance of uncertainty describes a tendency to respond negatively to uncertain situations or outcomes (Carleton et al., 2007). It has been associated with symptoms of anxiety and depression (Gentes and Ruscio, 2011), while its relationship with threat appraisal remains an area of theoretical and empirical debate (Milne et al., 2019). Judgments about another person's virtuous qualities can involve incomplete information about that person's intentions and behavior. We therefore considered intolerance of uncertainty a potentially relevant characteristic when examining such judgments. The cited research does not establish its relationship with perceptions of virtuous persons; the proposed negative association is examined in the present study.

Hopelessness involves pessimistic expectations about the future (Beck et al., 1974). Research on positive cognition has considered how future-oriented thoughts relate to psychological wellbeing (MacLeod and Moore, 2000). These findings raise the possibility that students' outlook on the future may also relate to how they evaluate desirable qualities in other people. Because the cited studies did not examine perceptions of virtuous persons, we treat the proposed negative association with hopelessness as a theoretical expectation to be tested in the present study.

Although virtue and character-related constructs have been extensively examined in the literature, empirical research specifically investigating perceptions of virtuous persons as a distinct psychological construct remains comparatively limited, particularly among university students. Existing studies have largely focused on virtue as an individual disposition or moral characteristic, with considerably less attention given to the psychological processes through which individuals perceive and evaluate virtuous others. Furthermore, although psychological wellbeing, resilience, intolerance of uncertainty, and hopelessness have each been associated with various aspects of psychological functioning, their combined contribution to perceptions of virtuous persons has not yet been examined within a unified predictive framework. Addressing this gap may contribute to a more comprehensive understanding of the psychological mechanisms underlying virtue-related evaluations during emerging adulthood. Accordingly, the present study aims to examine university students' perceptions of virtuous persons in relation to psychological wellbeing, psychological resilience, intolerance of uncertainty, and hopelessness.

In addition to the primary focus on psychological characteristics, we explored whether perceptions of virtuous persons varied across selected student characteristics. Gender, perceived income level, year of study, and academic achievement were included to describe variation within the university sample. Because the available literature does not provide a sufficiently specific basis for directional predictions about these differences in perceptions of virtuous persons, the demographic comparisons were treated as exploratory.

Based on the study objectives and the theoretical framework presented above, the following research question and hypotheses were formulated:

### 1.1 Research question

RQ1. Do perceptions of virtuous persons differ according to demographic characteristics, including gender, perceived income level, year of study, and academic achievement?

### 1.2 Hypotheses

H1. Psychological wellbeing is positively associated with perceptions of virtuous persons.

H2. Psychological resilience is positively associated with perceptions of virtuous persons.

H3. Intolerance of uncertainty is negatively associated with perceptions of virtuous persons.

H4. Hopelessness is negatively associated with perceptions of virtuous persons.

## 2 Method

The present study adopted a cross-sectional quantitative design using a correlational approach. The primary objective was to examine university students' perceptions of virtuous persons in relation to selected psychological variables and to explore whether these perceptions differed according to demographic characteristics. Within this framework, perceptions of virtuous persons constituted the dependent variable, whereas psychological wellbeing, psychological resilience, intolerance of uncertainty, and hopelessness were specified as predictor variables. Given the cross-sectional nature of the data, the analyses were designed to examine associations and predictive relationships rather than to support causal interpretations.

### 2.1 Participants

The study sample consisted of 1,322 undergraduate students aged 18 to 32 years who were enrolled at five universities located in Sivas, Sakarya, Samsun, Erzurum, and Gaziantep, Türkiye. University students were selected because higher education provides a socially and educationally diverse context in which individuals encounter different value systems and continuously evaluate interpersonal conduct, personal values, and future professional roles. Participants were recruited through institutional contacts using a non-probability convenience sampling strategy. Data were collected through an online questionnaire administered via Google Forms. The survey link was distributed to eligible students at the participating universities, and participation was entirely voluntary. This recruitment procedure facilitated access to students from different institutional and geographical contexts; however, the non-probability sampling method limits the generalizability of the findings.

Because the primary analyses involved multiple linear regression, particular attention was given to obtaining an adequate sample size for stable coefficient estimates. The final sample of 1,322 participants exceeded commonly recommended minimum sample size requirements for a regression model with four predictors (Hair et al., 2022; Tabachnick and Fidell, 2019). The inclusion of students from five universities located in different regions provided institutional and geographical diversity; however, the convenience sampling strategy limits the generalizability of the findings. Detailed demographic characteristics of the participants are presented in Table 1.

**Table 1:** Demographic characteristics of the study group.

Variable	Sub-variable	*n*	%
Gender	Male	287	21.7
	Female	1,035	78.3
University location	Sivas	245	18.5
	Sakarya	158	12.0
	Samsun	259	19.6
	Erzurum	304	23.0
	Gaziantep	356	26.9
Perceived income level	Low	524	39.6
	Medium	718	54.3
	High	80	6.1
Year of study	1. Class	363	27.5
	2. Class	374	28.3
	3. Class	295	22.3
	4. Class	290	21.9
Academic achievement	0–69	179	13.5
	70–74	192	14.5
	75–84	432	32.7
	85–89	349	26.4
	90–100	170	12.9

N = 1,322.

### 2.2 Data collection instruments

#### 2.2.1 Personal information form

A researcher-developed Personal Information Form was used to collect demographic data. The form included items assessing participants' age, gender, university location, perceived income level, year of study, and academic achievement. Gender, perceived income level, year of study, and academic achievement were included for exploratory comparisons within the undergraduate sample; no directional hypotheses were specified for these variables. Age and province of residence were collected to describe the sample.

#### 2.2.2 Virtuous person perception scale

The Virtuous Person Perception Scale, developed by Yigitce and Sentürk (2024), was used to assess individuals' perceptions of virtuous persons. The scale consists of 21 items distributed across five sub-dimensions: wisdom, chastity, generosity, justice, and courage. Items are rated on a five-point Likert scale, with higher scores indicating stronger perceptions of virtuous characteristics. In the present study, the internal consistency reliability coefficient (Cronbach's alpha) for the total scale was 0.81, indicating acceptable reliability. This value exceeds the commonly recommended threshold of 0.70 for research purposes, suggesting adequate internal consistency.

#### 2.2.3 Psychological wellbeing scale

Psychological wellbeing was assessed using the Psychological WellBeing Scale developed by Diener et al. (2010) and adapted into Turkish by Telef (2013). The scale consists of eight items and a single-factor structure. Responses are provided on a seven-point Likert scale, with higher scores reflecting greater psychological wellbeing and a stronger sense of meaning and purpose in life. In the current study, the Cronbach's alpha coefficient was 0.86, indicating good internal consistency.

#### 2.2.4 Short psychological resilience scale

Psychological resilience was measured using the Short Psychological Resilience Scale developed by Smith et al. (2010) and adapted into Turkish by Dogan (2015). The scale consists of six items and has a unidimensional structure. Items are rated on a five-point Likert scale, with higher scores indicating greater resilience. The internal consistency coefficient in the present sample was 0.82, demonstrating satisfactory reliability.

#### 2.2.5 Intolerance of uncertainty scale

The Intolerance of Uncertainty Scale, developed by Carleton et al. (2007) and adapted into Turkish by Sariçam et al. (2014), was used to assess individuals' responses to uncertain situations. The scale includes 12 items and two subscales: prospective anxiety and inhibitory anxiety. Higher scores indicate greater intolerance of uncertainty. In this study, the Cronbach's alpha coefficient for the total scale was 0.87, reflecting strong internal consistency.

#### 2.2.6 Beck hopelessness scale

Hopelessness was measured using the Beck Hopelessness Scale developed by Beck et al. (1974) and adapted into Turkish and psychometrically examined by Durak and Palabiyikoglu (1994). The scale consists of 20 dichotomous items (“Yes/No”). Higher total scores indicate greater levels of hopelessness. The internal consistency coefficient calculated for the present sample was 0.87, indicating good reliability.

### 2.3 Research process

Prior to data collection, ethical approval was obtained from the Ondokuz Mayis University Social and Human Sciences Research Ethics Committee (Decision No. 2024-883, dated September 27, 2024). Following ethical approval, data were collected via an online survey administered through Google Forms. The survey link was distributed to undergraduate students through institutional contacts and a mobile communication application. Participation was entirely voluntary. Before completing the questionnaires, participants were presented with an informed consent form outlining the purpose of the study, confidentiality assurances, and their right to withdraw at any time without penalty. Only those who provided consent were included in the study. The average time required to complete the survey instruments was approximately 20 min. Initially, 1,340 responses were obtained. Following data-quality screening, eight responses that did not meet the inclusion criteria because of substantial missing data or patterned/inconsistent responding were excluded. An additional 10 cases identified as extreme outliers were removed during statistical screening. Consequently, the final analyses were conducted with 1,322 participants. All data were collected and analyzed anonymously. No identifying information was recorded, and data were used exclusively for scientific purposes. Ethical principles, including confidentiality, voluntary participation, and responsible data management, were strictly observed throughout the research process.

### 2.4 Data analysis

Prior to statistical analyses, the data collected during the administration were merged into a single dataset. Responses that did not meet the inclusion criteria (*n* = 8) were excluded. The remaining data were entered into SPSS for analysis. Reverse-coded items were recoded before conducting statistical procedures. Data screening procedures were performed to ensure data quality. Outliers were examined using standardized z-scores and box plot inspections. Cases identified as extreme values that could adversely affect the dataset (*n* = 10) were removed. Consequently, the final analyses were conducted using data from 1,322 participants. Assumptions underlying parametric analyses were systematically evaluated. The normality assumption was assessed through histogram distributions and Q–Q plots. Additionally, skewness and kurtosis values were examined and found to fall within the acceptable range (±1.5), indicating approximate normal distribution. Reliability analyses were conducted to evaluate the internal consistency of the measurement instruments. Cronbach's alpha coefficients indicated acceptable reliability for all scales (α ≥ 0.70). Following assumption checks, descriptive statistics and bivariate relationships among the study variables were examined.

Descriptive statistics and bivariate correlations were calculated for the study variables. Exploratory differences in perceptions of virtuous persons were examined using an independent-samples *t*-test for gender and separate one-way analyses of variance (ANOVAs) for income level, year of study, and academic achievement. Tukey's HSD test was used for *post-hoc* comparisons when the omnibus ANOVA was statistically significant. Effect sizes were evaluated using Cohen's d for the gender comparison and eta squared (η^2^) for the one-way ANOVAs.

A multiple linear regression analysis was conducted to examine the unique associations of psychological wellbeing, psychological resilience, intolerance of uncertainty, and hopelessness with perceptions of virtuous persons. All four predictors were entered simultaneously using the Enter method. Linearity was evaluated through scatterplots, residual independence through the Durbin–Watson statistic, and multicollinearity through tolerance and variance inflation factor (VIF) values. Visual inspection of the residual plots indicated no substantial violations of linearity, homoscedasticity, or residual normality. The Durbin–Watson value was 1.966, while tolerance values ranged from 0.679 to 0.810 and VIF values ranged from 1.234 to 1.473, indicating no evidence of residual dependence or problematic multicollinearity. All analyses were conducted using SPSS, and statistical significance was set at *p* < 0.05.

## 3 Findings

Findings related to descriptive analysis and regression analysis are presented below in sequence.

To address RQ1, an independent-samples *t*-test was conducted to examine whether perceptions of virtuous persons differed by gender. Levene's test was statistically significant, *F* = 6.32, *p* = 0.012; therefore, the results based on unequal variances were interpreted. Female students (*M* = 80.77, *SD* = 9.24) reported significantly higher perceptions of virtuous persons than male students (*M* = 78.71, *SD* = 10.10), Welch's *t*_(428.03)_ = 3.11, *p* = 0.002, 95% CI [0.76, 3.36]. However, the magnitude of the difference was small (Cohen's *d* = 0.22). The results are presented in Table 2.

**Table 2:** Independent samples *t*-test results for perceptions of virtuous persons by gender.

Variable	Gender	*n*	*M*	*SD*	*t(df)*	*p*	95% CI for mean difference	Cohen's *d*
Perceptions of virtuous persons	Female	1,035	80.77	9.24	3.11 (428.03)	0.002	(0.76, 3.36)	0.22
	Male	287	78.71	10.10				

N = 1,322. Because Levene's test was statistically significant, F = 6.32, *p* = 0.012, results based on unequal variances (Welch's t-test) are reported. The effect size was small (d = 0.22).

As presented in Table 3, one-way ANOVA results indicated statistically significant differences in perceptions of virtuous persons across perceived income level, year of study, and academic achievement. Perceived income level yielded a statistically significant difference, *F*_(2, 1319)_ = 6.812, *p* = 0.001, η^2^ = 0.010, with students in the low-income group reporting lower scores than those in the medium- and high-income groups. Year of study also yielded a statistically significant difference, *F*_(3, 1318)_ = 7.386, *p* < 0.001, η^2^ = 0.017, with fourth-year students reporting higher scores than first-, second-, and third-year students. Academic achievement produced a statistically significant difference, *F*_(4, 1317)_ = 9.565, *p* < 0.001, η^2^ = 0.028, with students in the 0–69 achievement group reporting lower scores than all other groups. However, all effect sizes were small according to Cohen's (1988) criteria, indicating that these demographic characteristics accounted for only limited variation in perceptions of virtuous persons.

**Table 3:** One-way ANOVA results for perceptions of virtuous persons by perceived income level, year of study, and academic achievement.

Variable	Group	*n*	*M*	*SD*	*F* (df1, df2)	*p*	*η^2^*	*Post-hoc* (Tukey)
Perceived income Level	Low	524	79.25	10.04	6.812 (2, 1,319)	0.001	0.010	Low < Medium, High
	Medium	718	80.86	9.06				
	High	80	82.56	8.38				
Year of study	1st	363	79.38	9.41	7.386 (3, 1,318)	< 0.001	0.017	4th > 1st, 2nd, 3rd
	2nd	374	79.80	9.19				
	3rd	295	79.92	9.58				
	4th	290	82.59	9.46				
Academic achievement	0–69	179	76.55	10.52	9.565 (4, 1,317)	< 0.001	0.028	0–69 < all
	70–74	192	79.65	9.17				
	75–84	432	81.00	9.12				
	85–89	349	81.19	9.27				
	90–100	170	81.32	8.95				

N = 1,322. M, mean; SD, standard deviation; df, degrees of freedom. Effect sizes (η^2^) were calculated using the formula η^2^ = (df_between × F) / (df_between × F + df_within). According to Cohen (1988), η^2^ values of 0.01, 0.06, and 0.14 represent small, medium, and large effect sizes, respectively. Post-hoc comparisons were conducted using Tukey's HSD test.

A multiple linear regression analysis was conducted to examine the unique associations of psychological wellbeing, psychological resilience, intolerance of uncertainty, and hopelessness with perceptions of virtuous persons. The results are presented in Table 4.

**Table 4:** Multiple linear regression predicting perceptions of virtuous persons.

Predictor	*B*	*SE*	β	*t*	*p*	95% CI for B	Tolerance	VIF
Psychological wellbeing	0.692	0.031	0.580	22.146	< 0.001	[0.631, 0.753]	0.709	1.410
Psychological resilience	0.049	0.050	0.024	0.975	0.330	[−0.050, 0.147]	0.791	1.263
Intolerance of uncertainty	0.143	0.025	0.138	5.618	< 0.001	[0.093, 0.192]	0.810	1.234
Hopelessness	−0.038	0.057	−0.018	−0.675	0.500	[−0.150, 0.073]	0.679	1.473

N = 1,322. Dependent variable: perceptions of virtuous persons. R = 0.599, R^2^ = 0.359, adjusted R^2^ = 0.357, F_(4, 1, 317)_ = 184.580, *p* < 0.001, Durbin–Watson = 1.966. B, unstandardized coefficient; SE, standard error; β, standardized coefficient; CI, confidence interval; VIF, variance inflation factor.

The multiple regression model was statistically significant, *F*_(4, 1317)_ = 184.580, *p* < 0.001, and explained 35.9% of the variance in perceptions of virtuous persons (*R*^2^ = 0.359; adjusted *R*^2^ = 0.357). Psychological wellbeing was the strongest positive predictor (β = 0.580, *p* < 0.001), supporting H1. Intolerance of uncertainty also made a significant positive contribution (β = 0.138, *p* < 0.001); however, this association was contrary to the negative direction proposed in H3. Psychological resilience (β = 0.024, *p* = 0.330) and hopelessness (β = −0.018, *p* = 0.500) did not make significant unique contributions to the model. Accordingly, H2, H3, and H4 were not supported. Tolerance values ranged from 0.679 to 0.810 and VIF values ranged from 1.234 to 1.473, indicating no problematic multicollinearity among the predictors.

## 4 Conclusion and discussion

Exploratory analyses indicated that female students reported somewhat higher perceptions of virtuous persons than male students. Perceptions also differed across income groups. Although gender-related socialization and access to social or educational resources could be considered in future research, neither was measured in the present study. The observed group differences therefore do not establish why students' scores varied. The income difference was small (η^2^ = 0.010), and its practical significance should be interpreted cautiously.

Perceptions of virtuous persons also differed by year of study and academic achievement. Fourth-year students reported higher scores than students in earlier years, but the effect was small (η^2^ = 0.017). Because different students were compared at a single point in time, this finding does not show that perceptions change as students progress through university. Similarly, the difference across academic achievement groups was small (η^2^ = 0.028). The study did not assess learning engagement, reflective thinking, or self-regulation as explanations for this difference. Longitudinal research and direct measures of these processes would be needed to examine the proposed educational and psychological interpretations.

Taken together, the demographic comparisons describe modest variation in perceptions of virtuous persons within this sample. They should be viewed as exploratory findings rather than evidence that demographic characteristics or university experiences determine these perceptions. We next consider the associations between perceptions of virtuous persons and the psychological variables that formed the primary focus of the study.

Psychological wellbeing emerged as the strongest positive predictor of university students' perceptions of virtuous persons, even after psychological resilience, intolerance of uncertainty, and hopelessness were included in the model. This finding supports H1 and is consistent with the theoretical expectation that positive psychological functioning may be associated with more constructive value-based evaluations. Individuals with higher psychological wellbeing typically report greater meaning in life, psychological integration, and positive interpersonal functioning (Ryff and Singer, 1996; Kesebir and Diener, 2014). These characteristics may provide a psychological context in which virtuous qualities are more readily recognized and positively evaluated. Previous research has also linked psychological wellbeing with adaptive cognitive functioning, constructive interpersonal relationships, and positive future-oriented cognition (Liu et al., 2019; MacLeod and Moore, 2000). The present finding extends this literature by showing that psychological wellbeing has a substantial unique association with perceptions of virtuous persons within the combined regression model. Nevertheless, because the study was cross-sectional, the result does not establish that psychological wellbeing causes more positive virtue-related perceptions. Longitudinal research is needed to determine the direction and development of this association.

Contrary to H2, psychological resilience did not make a significant unique contribution to perceptions of virtuous persons when psychological wellbeing, intolerance of uncertainty, and hopelessness were included in the regression model (β = 0.024, *p* = 0.330). Although psychological resilience showed a small positive bivariate correlation with perceptions of virtuous persons (*r* = 0.114, *p* < 0.001), this association was no longer significant after the other psychological variables were controlled. This pattern suggests that the initial association may largely reflect variance shared with the other predictors rather than an independent contribution of resilience. Previous research has associated resilience with adaptive coping, emotional regulation, and the maintenance of psychological functioning under adversity (Rutter, 1985; Tugade et al., 2004; Davydov et al., 2010; Sisto et al., 2019). However, these established functions of resilience do not necessarily translate into a unique association with how individuals perceive virtuous qualities in others. Accordingly, the present findings do not support the hypothesis that psychological resilience independently predicts perceptions of virtuous persons. Future research could examine whether resilience is related to virtue-related perceptions indirectly through psychological wellbeing, emotional regulation, or other unmeasured processes.

Contrary to H3, intolerance of uncertainty made a significant positive contribution to perceptions of virtuous persons when the other psychological variables were controlled (β = 0.138, *p* < 0.001). Its bivariate correlation with perceptions of virtuous persons was also positive, although small (*r* = 0.083, *p* = 0.001). This finding differs from the hypothesized negative association, which was based on literature linking intolerance of uncertainty with threat appraisal, psychological symptoms, and difficulty responding adaptively to ambiguous situations (Gentes and Ruscio, 2011; Milne et al., 2019; Knowles and Olatunji, 2023). However, these previous studies did not examine perceptions of virtuous persons or value-based evaluations. One tentative interpretation is that individuals who are less comfortable with uncertainty may rely more strongly on clearly defined virtuous characteristics as stable standards for evaluating others. They may therefore endorse qualities such as justice, wisdom, generosity, courage, and chastity more strongly because these qualities provide relatively clear normative reference points. Nevertheless, the present study did not measure moral rigidity, need for certainty, or the cognitive processes involved in interpersonal evaluation. The positive association should therefore be interpreted cautiously and requires replication and direct examination in future research.

H4 was not supported because hopelessness did not make a significant unique contribution to perceptions of virtuous persons after the other psychological variables were included in the regression model (β = −0.018, *p* = 0.500). At the bivariate level, hopelessness was moderately and negatively correlated with perceptions of virtuous persons (*r* = −0.291, *p* < 0.001). However, hopelessness was also substantially associated with psychological wellbeing (*r* = −0.519, *p* < 0.001), suggesting that its bivariate association with perceptions of virtuous persons may largely reflect variance shared with psychological wellbeing and the other predictors. Hopelessness is generally characterized by pessimistic expectations about the future (Beck et al., 1974), while future-oriented cognitions have been linked to psychological wellbeing and mental health (MacLeod and Moore, 2000). Nevertheless, the present results do not support the hypothesis that hopelessness is independently associated with perceptions of virtuous persons. Future studies could examine whether hopelessness is related to virtue-related perceptions indirectly through psychological wellbeing or other cognitive and motivational processes.

Overall, the present study contributes to the literature by examining perceptions of virtuous persons as value-related evaluations that may be associated with psychological functioning as well as contextual characteristics. The findings showed that the psychological variables collectively explained a substantial proportion of variance in perceptions of virtuous persons; however, their unique contributions were not uniform. Psychological wellbeing emerged as the strongest positive predictor, whereas intolerance of uncertainty showed a smaller positive association, contrary to the hypothesized direction. Psychological resilience and hopelessness did not make significant unique contributions after the other predictors were considered. The exploratory demographic findings also indicated only modest differences within the sample. Accordingly, the results support a more differentiated account of virtue perception rather than a general distinction between protective resources and vulnerability-related factors. Future research should examine these relationships using longitudinal and cross-cultural designs and directly assess the cognitive, emotional, and evaluative processes that may account for the observed associations.

## 5 Implications

The findings have theoretical implications for educational psychology and higher education by showing the value of examining perceptions of virtuous persons in relation to psychological functioning. Rather than treating the perception of virtue exclusively as a moral or philosophical judgment, the present study approaches it as a value-related evaluative process that may also be associated with individual psychological characteristics. The simultaneous examination of the four predictors revealed that these characteristics did not operate uniformly: psychological wellbeing showed the strongest unique association, intolerance of uncertainty showed a smaller positive association, contrary to expectations, and psychological resilience and hopelessness did not make significant unique contributions. This pattern challenges a simple distinction between protective psychological resources and vulnerability-related factors and indicates that their relationships with virtue perception may be more differentiated than originally proposed. Within the higher education context, the findings provide an initial basis for investigating how students' psychological functioning relates to the standards they use when recognizing and evaluating virtuous qualities.

From a practical perspective, the strong association between psychological wellbeing and perceptions of virtuous persons suggests that student wellbeing and value-related reflection may be meaningfully considered together within higher education. Universities could incorporate opportunities for ethical reflection, discussion of moral exemplars, and value clarification into existing student development and wellbeing initiatives. However, the present findings do not establish that interventions designed to improve psychological wellbeing will produce changes in perceptions of virtuous persons. They also do not provide sufficient support for recommending resilience-, hope-, or uncertainty-focused programs as methods for modifying virtue-related perceptions, because psychological resilience and hopelessness did not make significant unique contributions, and intolerance of uncertainty was associated with the outcome in an unexpected positive direction. Intervention and longitudinal studies are therefore needed before specific practical programs can be recommended on the basis of these relationships.

## 6 Limitations and suggestions

Despite its contributions, this study has several limitations. First, its cross-sectional design does not permit causal or temporal conclusions about the relationships between psychological characteristics and perceptions of virtuous persons. Terms such as predictor in the present study refer to statistical prediction rather than causation. Longitudinal and experimental studies are needed to determine whether changes in psychological functioning precede changes in virtue-related perceptions. Second, all variables were assessed using self-report instruments administered as part of the same data-collection procedure. The findings may therefore be affected by shared method variance, social desirability, and participants' response tendencies. Future research could combine self-report measures with behavioral tasks, judgments of moral scenarios, peer reports, or qualitative interviews.

Third, participants were recruited via convenience sampling from five universities in Türkiye, and female students constituted the majority of the sample. These characteristics limit the sample's representativeness and the generalizability of the findings to other student populations, educational settings, and cultural contexts. Fourth, the study did not directly assess the cognitive and evaluative mechanisms proposed in interpreting the findings, such as moral certainty, need for closure, ethical reflection, or value internalization. This limitation is particularly relevant to the unexpected positive association between intolerance of uncertainty and perceptions of virtuous persons. The proposed explanations should therefore be regarded as tentative until they are examined using direct measures and replicated in independent samples.

## Data Availability

The data analyzed in this study is subject to the following licenses/restrictions: the data that support the findings of this study are available on request from the corresponding author. Requests to access these datasets should be directed to dgur@sakarya.edu.tr.

## References

[B1] Arnett J. J. (2000). Emerging adulthood: a theory of development from the late teens through the twenties. Am. Psychol. 55, 469–480. doi: 10.1037/0003-066X.55.5.469 10842426

[B2] Arnett J. J. (2007). Emerging adulthood: what is it, and what is it good for? Child Dev. Perspect. 1, 68–73. doi: 10.1111/j.1750-8606.2007.00016.x

[B3] Beck A. T. Weissman A. Lester D. Trexler L. (1974). The measurement of pessimism: the Hopelessness Scale. J. Consult. Clin. Psychol. 42, 861–865. doi: 10.1037/h0037562 4436473

[B4] Carleton R. N. Norton M. A. P. J. Asmundson G. J. G. (2007). Fearing the unknown: a short version of the Intolerance of Uncertainty Scale. J. Anxiety Disord. 21, 105–117. doi: 10.1016/j.janxdis.2006.03.014 16647833

[B5] Carr D. (2017). Virtue and character in higher education. Bri. J. Educ. Stud. 65, 109–124. doi: 10.1080/00071005.2016.1224806

[B6] Cohen J. (1988). Statistical Power Analysis for the Behavioral Sciences, 2nd Edn. Hillsdale, NJ: Lawrence Erlbaum Associates.

[B7] Daniilidou A. Nerantzaki K. (2025). Character virtues in romantic relationships and friendships during emerging adulthood: a latent profile approach. Psychol. Int. 7, 1–21. doi: 10.3390/psycholint7040091

[B8] Davydov D. M. Stewart R. Ritchie K. Chaudieu I. (2010). Resilience and mental health. Clin. Psychol. Rev. 30, 479–495. doi: 10.1016/j.cpr.2010.03.003 20395025

[B9] Diener E. Wirtz D. Tov W. Kim-Prieto C. Choi D. W. Oishi S. . (2010). New well-being measures: short scales to assess flourishing and positive and negative feelings. Soc. Indic. Res. 97, 143–156. doi: 10.1007/s11205-009-9493-y

[B10] Dogan T. (2015). Kisa psikolojik saglamlik ölçegi'nin Türkçe uyarlamasi: geçerlik ve güvenirlik çalişmasi. J. Happiness Well-Being 3, 93–102.

[B11] Durak A. Palabiyikoglu R. (1994). Beck Umutsuzluk Ölçegi geçerlilik çalişmasi. Kriz Derg. 2, 311–319. doi: 10.1501/0000831

[B12] Fortenbaugh W. W. (1964). “Aristotle's conception of moral virtue and its perceptive role,” in Transactions and Proceedings of the American Philological Association, Vol. 95 (Baltimore, MD: Johns Hopkins University Press, American Philological Association), 77–87.

[B13] Gentes E. L. Ruscio A. M. (2011). A meta-analysis of the relation of intolerance of uncertainty to symptoms of generalized anxiety disorder, major depressive disorder, and obsessive-compulsive disorder. Clin. Psychol. Rev. 31, 923–933. doi: 10.1016/j.cpr.2011.05.001 21664339

[B14] Gyamera G. O. (2018). I just want to be somebody: exploring students' Motivations and experiences in universities. South Afr. J. High. Educ. 32, 154–171. doi: 10.20853/32-3-2544

[B15] Hair J. F. Black W. C. Babin B. J. Anderson R. E. (2022). Multivariate Data Analysis, 9th Edn. Andover: Cengage.

[B16] Holahan C. (2012). Environment and Behavior: A Dynamic Perspective. New York, NY: Springer Science and Business Media.

[B17] Jacobson D. (2005). Seeing by feeling: virtues, skills, and moral perception. Ethical Theory Moral Pract. 8, 387–409. doi: 10.1007/s10677-005-8837-1

[B18] Kesebir P. Diener E. (2014). “A virtuous cycle: the relationship between happiness and virtue,” in The Philosophy and Psychology of Character and Happiness (New York, NY: Routledge), 287–306.

[B19] Knowles K. A. Olatunji B. O. (2023). Intolerance of uncertainty as a cognitive vulnerability for obsessive-compulsive disorder: a qualitative review. Clin. Psychol. Sci. Pract. 30, 317–330. doi: 10.1037/cps0000150 39431164 PMC11488751

[B20] Kong T. Zeng S. (2023). The effect of perceived environmental uncertainty on university students' anxiety, academic engagement, and prosocial behavior. Behav. Sci. 13, 1–14. doi: 10.3390/bs13110906 37998653 PMC10669797

[B21] Kristiansen C. M. Hotte A. M. (2013). “Morality and the self: implications for the when and how of value-attitude-behavior relations,” in The Psychology of Values (New York, NY: Psychology Press), 77–105.

[B22] Kristjánsson K. (2015). Aristotelian Character Education. London: Routledge.

[B23] Kunkel J. H. (2021). “The behavioral perspective of social dynamics,” in Behavioral Theory in Sociology (London: Routledge), 433–468.

[B24] Lickona T. (2013). “Character education: the cultivation of virtue,” in Instructional-Design Theories and Models (New York, NY: Routledge), 591–612.

[B25] Liu S. Wang P. Gao W. Song M. Zhang N. (2025). Virtuous personality and bystander defending behavior among college students: roles of moral identity and friendship quality. BMC Psychol. 13, 1–12. doi: 10.1186/s40359-025-03058-4 40616135 PMC12228306

[B26] Liu X. Ping S. Gao W. (2019). Changes in undergraduate students' psychological well-being as they experience university life. Int. J. Environ. Res. Public Health 16:2864. doi: 10.3390/ijerph16162864 31405114 PMC6719208

[B27] MacLeod A. K. Moore R. (2000). Positive thinking revisited: positive cognitions, well-being and mental health. Clin. Psychol. Psychotherapy 7, 1–10. doi: 10.1002/(SICI)1099-0879(200002)7:1<1::AID-CPP228>3.0.CO;2-S

[B28] Milne S. Lomax C. Freeston M. H. (2019). A review of the relationship between intolerance of uncertainty and threat appraisal in anxiety. Cogn. Behav. Ther. 12:e38. doi: 10.1017/S1754470X19000230

[B29] Mitchell L. A. (2015). Integrity and virtue: the forming of good character. Linacre Q. 82, 149–169. doi: 10.1179/2050854915Y.0000000001 25999613 PMC4434789

[B30] Moberg D. J. (2000). Role models and moral exemplars: how do employees acquire virtues by observing others?. Bus. Ethics Q. 10, 675–696. doi: 10.2307/3857898

[B31] Niemiec R. M. (2018). Character Strengths Interventions: A Field Guide for Practitioners. Boston, MA: Hogrefe.

[B32] Ossowska M. (2016). Social Determinants of Moral Ideas. Philadelphia, PA: University of Pennsylvania Press.

[B33] Peterson C. Seligman M. E. P. (2004). Character Strengths and Virtues: A Handbook and Classification. New York, NY: American Psychological Association; Oxford University Press.

[B34] Rutter M. (1985). Resilience in the face of adversity: protective factors and resistance to psychiatric disorder. Br. J. Psychiatry 147, 598–611. doi: 10.1192/bjp.147.6.598 3830321

[B35] Ryff C. D. Singer B. (1996). Psychological well-being: meaning, measurement, and implications for psychotherapy research. Psychother. Psychosom. 65, 14–23. doi: 10.1159/000289026 8838692

[B36] Sariçam H. Erguvan F. M. Akin A. Akça M. S. (2014). The Turkish short version of the intolerance of uncertainty (IUS-12) scale: the study of validity and reliability. Route Educ. Soc. Sci. J. 1, 148–157. doi: 10.17121/ressjournal.109

[B37] Scanlon L. Rowling L. Weber Z. (2007). ‘You don't have like an identity… you are just lost in a crowd': forming a student identity in the first-year transition to university. J. Youth Stud. 10, 223–241. doi: 10.1080/13676260600983684

[B38] Sentürk S. Altinişik M. S. Altun A. Koçyigit Özbay G. (2024). Lise ögrencilerinin erdemli insan algilarinin çeşitli degişkenler açisindan incelenmesi [An examination of high school students' perceptions of virtuous persons in terms of various variables]. Degerler Egit. Derg. 22, 465–498. [In Turkish]. doi: 10.34234/ded.1540150

[B39] Sisto A. Vicinanza F. Campanozzi L. L. Ricci G. Tartaglini D. Tambone V. (2019). Towards a transversal definition of psychological resilience: a literature review. Medicina 55:745. doi: 10.3390/medicina55110745 31744109 PMC6915594

[B40] Smith B. W. Tooley E. M. Christopher P. J. Kay V. S. (2010). Resilience as the ability to bounce back from stress: a neglected personal resource?. J. Posit. Psychol. 5, 166–176. doi: 10.1080/17439760.2010.482186

[B41] Sparks P. Shepherd R. (2002). The role of moral judgments within expectancy-value-based attitude-behavior models. Ethics Behav. 12, 299–321. doi: 10.1207/S15327019EB1204_01

[B42] Tabachnick B. G. Fidell L. S. (2019). Using Multivariate Statistics, 7th Edn. New York, NY: Pearson.

[B43] Telef B. B. (2013). Psikolojik iyi oluş ölçegi: Türkçeye uyarlama, geçerlik ve güvenirlik çalişmasi. Hacet. Üniv. Egit. Fak. Derg. 28, 374–384.

[B44] Torres V. Jones S. R. Renn K. A. (2009). Identity development theories in student affairs: origins, current status, and new approaches. J. Coll. Stud. Dev. 50, 577–596. doi: 10.1353/csd.0.0102

[B45] Tugade M. M. Fredrickson B. L. Feldman Barrett L. (2004). Psychological resilience and positive emotional granularity: examining the benefits of positive emotions on coping and health. J. Pers. 72, 1161–1190. doi: 10.1111/j.1467-6494.2004.00294.x 15509280 PMC1201429

[B46] Turan R. (2015). Ibn miskeveyh'de erdem kavrami ve temel erdemler. Namik Kemal Üniv. Ilah. Fak. Derg. 1, 7–35.

[B47] Vallor S. (2012). Flourishing on facebook: virtue friendship and new social media. Ethics Inf. Technol. 14, 185–199. doi: 10.1007/s10676-010-9262-2

[B48] Walker L. J. Frimer J. A. (2007). Moral personality of brave and caring exemplars. J. Pers. Soc. Psychol. 93, 845–860. doi: 10.1037/0022-3514.93.5.845 17983304

[B49] Williams B. (2018). “Acting as the virtuous person acts,” in Aristotle and Moral Realism (London: Routledge), 13–33.

[B50] Yigitce A. N. Sentürk S. (2024). Erdemli insan algisi ölçegi: geçerlik ve güvenilirlik çalişmasi [Virtuous person perception scale: a validity and reliability study]. Ondokuz Mayis Üniv. Egit. Fak. Derg. 43, 397–449. [In Turkish]. doi: 10.7822/omuefd.1334877

